# PRODUCT REVIEW / ÉVALUATION DE PRODUIT

**DOI:** 10.29173/jchla29877

**Published:** 2025-08-01

**Authors:** Angélique Roy, BHums

**Affiliations:** Health Sciences Librarian Queen’s University Kingston, ON, Canada

**Product:** Perplexity

**URL:** https://www.perplexity.ai/

## Purpose

Perplexity combines large language models (LLMs) with live web search to provide current, sourced answers rather than generating text from stored knowledge. It specializes in real-time, citation-backed research as opposed to serving as a conversational partner [1].

## Product description

Perplexity is a generative AI (artificial intelligence) powered search engine that uses machine learning and natural language processing to provide thorough answers to queries by searching the web for trusted sources and then synthesizing relevant information into a detailed response. Unlike traditional search engines which provide dozens of pages of links to explore individually, Perplexity provides answers in sentence form instantaneously [2]. Conversations are stored as Threads which include the initial question and all follow-up queries and responses.

**Fig. 1:**
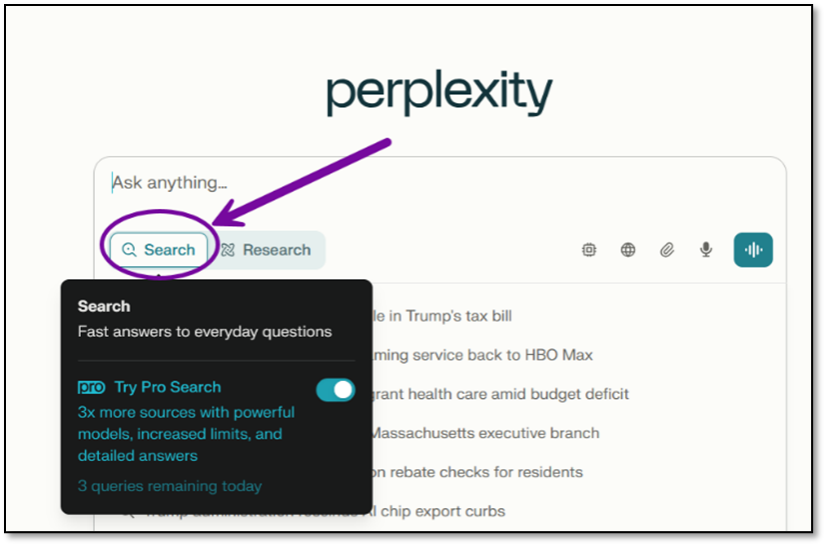
Search mode

There are two modes to choose from in the free version: Search and Research. Search is the default setting, designed for quicker and more concise responses to general questions (Figure 1).

**Fig. 2:**
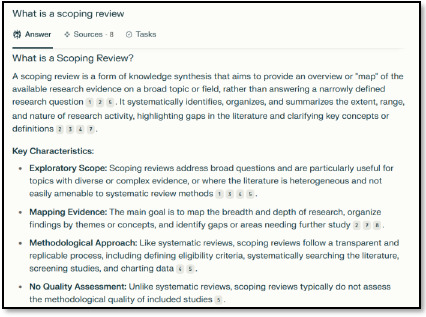
Sample search response

When using the Search function, a response starts generating immediately (Figure 2).

**Fig. 3:**
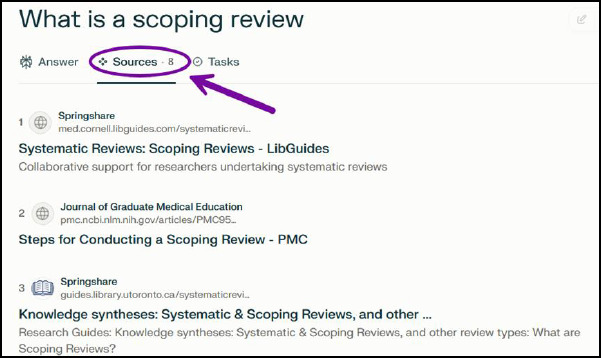
Search mode Sources tab

**Fig. 4:**
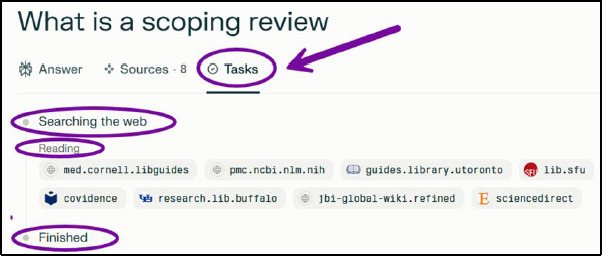
Search mode Tasks tab

Once a response is generated, a tab for Sources and a tab for Tasks appear. Clicking on Sources shows a list of all sources cited within the response (Figure 3) and clicking on Tasks provides an outline of what the AI did to retrieve the answer (Figure 4).

Citations are typically retrieved from a variety of websites and can also include library research guides, preprint servers, and other discipline-specific sources. It is possible journal articles will be included, but Research mode is preferable for retrieving scholarly works as it executes multiple searches and gathers information from across hundreds of sources [3]. Users also have the option to hover over Search and toggle the Pro version on to receive three Pro Search queries per day without upgrading to a paid subscription. Pro Search provides more detailed responses than Search mode by using multi-step reasoning to break down complex searches or prompts into steps for the AI to synthesize sources.

**Fig. 5:**
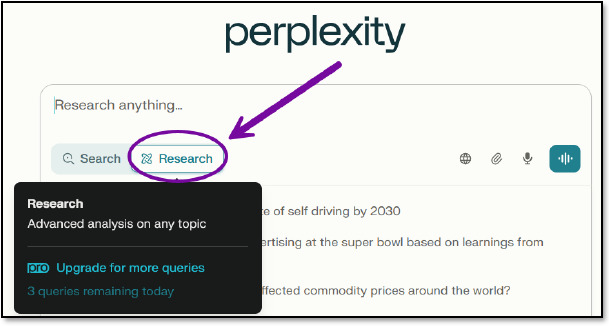
Research mode

Research mode is intended for questions requiring longer and more in-depth responses and can be used up to three times per day with the free version (Figure 5).

**Fig. 6:**
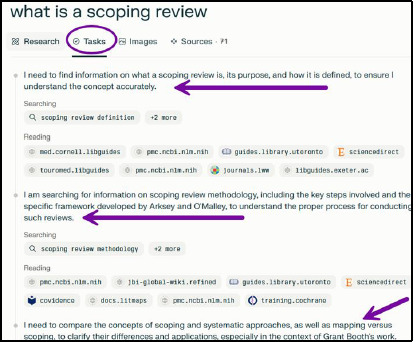
Research mode Tasks tab

When using the Research function, the AI explains each step of the process in first person statements as it searches and reads for information to answer the query. Once complete, this information can be found in the Tasks tab (Figure 6).

The list of sources is more extensive than that of Search mode with academic literature more prevalent, and may take upwards of five minutes to generate the comprehensive response [3].

**Fig. 7:**
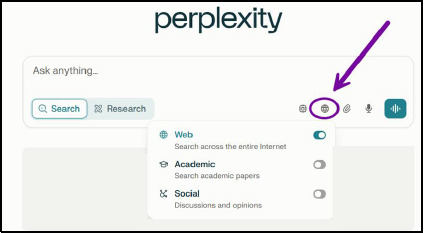
Search sources selection

In both modes, images and videos may also be retrieved depending on the question asked by the user and will be stored in the separate Images and Videos tabs. Free and paid account users can set the Search mode to search the Internet (default setting), academic papers, or social media discussions and opinions (Figure 7).

**Fig. 8:**
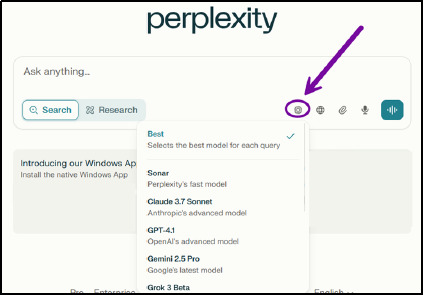
LLM selection

Perplexity works with both prompts and searches. Prompts are detailed, conversational commands, whereas searches are a set of keywords like those used in a search engine. Keywords can also be combined using Boolean operators (AND, OR, NOT). Prompts should be used when seeking more detailed explanations or specific content while searches are best for yielding broader results [4]. While both prompts and searches generate responses in paragraph format, a prompt response will be more in-depth. The default large language model (LLM) for searches is set to “Best” where the system chooses the most appropriate LLM for the query. Pro subscription users can choose from a list of eight additional LLMs including Perplexity’s fast model, Sonar, as well as other major models like Claude 3.7 Sonnet (Anthropic’s advanced model), GPT-4.1 (OpenAI’s advanced model), and Gemini 2.5 Pro (Google’s latest model), as well as more advanced reasoning models such as R1 1776 (Perplexity’s self-proclaimed unbiased reasoning model, see Figure 8) [1].

## Intended users

Perplexity is intended for anyone conducting research from basic searches to advanced prompts. By citing sources that users can review and evaluate for themselves, it positions itself as a search engine that provides trustworthy answers.

## Special features

The free version of Perplexity includes a variety of features and functions that may be advantageous for users based on preferred modes of information gathering. These include but are not limited to:

- 10 text, audio, or video file uploads per day: upload from device, Google Drive, or Dropbox to receive summaries, transcription, brainstorming, coding, and more [5]
- Image generation: use prompts to generate images (browser only as of May 2025)
- Discover: browse topics from finance to entertainment and customize them according to personal preferences
- Spaces: create a Space and give the AI instructions that apply to every thread within it
- Dictation: dictate a query rather than typing it
- Voice Mode: ask questions by voice and the AI will respond orally
- Set your preferred language: select from a list of 21 available languages to be used in the interface and responses
- Appearance: choose between light and dark modes.

## Platform and compatibility

Perplexity works in any Internet browser. It has a desktop app for Mac and Windows and an iOS and Android app for mobile. In April 2025, Perplexity CEO and co-founder, Aravind Srinivas, announced Perplexity’s integration with the WhatsApp messaging platform owned by Meta [6]. As of May 2025, there is a waitlist for Perplexity Comet, an agentic browser that is automated to act on the user’s behalf through natural language instructions [1].

## Usability

To access Perplexity’s primary features, users must create an account and sign in with Google, Apple, or email. The interface is intuitive and can be used across the web and desktop or mobile apps based on user preference.

## Strengths and weaknesses

### Strengths

- Provides at least eight in-text citations for each query
- Pulls information from the web in real time
- Includes options to search the web or academic sources
- Leverages multiple LLMs in addition to proprietary models

### Weaknesses

- Information accuracy is not guaranteed so users are encouraged to review sources [2,4]
- Lack of transparency around source selection process, potential for hallucinations, and bias
- Choice of LLM is exclusive to Pro users
- Search Pro and Research mode have daily limits for free accounts

## Privacy

Perplexity features various advanced options for managing privacy. Under My Account, users have options to sign out of sessions across all devices or browsers or to permanently delete their account and data. Data is collected from devices and interactions on the website, but personal data is not sold or shared with third parties. Under Preferences, the AI data retention setting can be turned off to disable Perplexity from using searches within the account to improve AI models [7].

## Cost

The free account includes unlimited basic searches and a limited number of Pro searches and Research queries daily. The Pro version is $20 per month or $200.04 when billed annually (USD). Pro includes unlimited access to Pro Search and Perplexity Research as well as unlimited uploads. Pro users can choose from eight LLMs [1]. Perplexity also offers an Enterprise Pro subscription as a platform integration solution for businesses.

## Comparison with similar products

With the proliferation of AI tools, there are several similar AI-powered search engines that may be valuable to researchers. Table 1 below identifies Consensus and Elicit as comparable tools with free and subscription account options.

**Table 1:** Comparison of Perplexity with similar AI search engines

AI Search Engine	Primary Purpose	SourceGeneration	Unique Features	Subscription Levels (USD)
Perplexity	General knowledge and deep research search engine	Real-time web search for citations	Real-time web searching	Pro: $20/month or $200.04/year Enterprise Pro plan available
Consensus	Scientific research search engine	Semantic Scholar database	Academic literature focus Consensus Meter for yes/no questions	Premium: $11.99/monthor $107.88/year Teams Plan and Enterprise Plan available
Elicit	Academic research assistant	Semantic Scholar database	Academic literature focus Systematic Review workflow (Pro only)	Elicit Plus:$12/month or $120/year Elicit Pro: $49/month or$499/year Elicit Team planavailable

## Conclusion

Perplexity combines web searching with large language models to provide comprehensive research responses. Its inclusion of in-text citations allows users to verify information for accuracy and credibility. While several other AI-powered search engines focus on academic literature, Perplexity serves as an effective general-purpose research tool for various users. The platform bridges traditional search engines and AI assistants by offering both synthesized answers and source attribution.
